# Effectiveness of acupressure in managing nausea, vomiting, and psychosocial well-being during pregnancy

**DOI:** 10.1590/1980-220X-REEUSP-2026-0082en

**Published:** 2026-07-24

**Authors:** Yeliz Doğan Merih, Zehra Acar, Özlem Karabulut, Mehlika Aslan

**Affiliations:** 1 University of Health Sciences, Hamidiye Faculty of Nursing, Istanbul, Türkiye.; 2 Independent Researcher, Istanbul, Türkiye.; 3 Istanbul Univesity, Faculty of Nursing, Istanbul, Türkiye.

**Keywords:** Acupressure, Efficacy, Nausea, Pregnancy, Vomiting, Wrist, Acupressão, Eficácia, Náusea, Gravidez, Vômito, Punho

## Abstract

**Objective:**

To examine the effectiveness of acupressure applied to the P6 point in reducing nausea and vomiting during pregnancy and its influence on psychosocial well-being.

**Method:**

This quasi-experimental pretest–posttest study included 61 pregnant women (31 intervention, 30 control). Data were collected using the Pregnancy Descriptive Information Form, the Pregnancy-Unique Quantification of Emesis (PUQE) Test, and the Pregnancy Psychosocial Health Assessment Scale (PPHAS).

**Results:**

Following the intervention, the acupressure wristband group demonstrated a significantly greater reduction in PUQE scores compared to the placebo group. Repeated measures analysis showed a significant and progressive decrease in nausea and vomiting severity in the acupressure wristband group from baseline through the fifth day, whereas no significant change was observed in the placebo group. PPHAS scores increased significantly within both groups after the intervention, however, no statistically significant difference was found between groups in post-intervention scores.

**Conclusion:**

P6 acupressure appears to be a safe, practical, and non-pharmacological method that effectively reduces nausea and vomiting and may support psychosocial well-being in early pregnancy.

## RESUMO

**Objetivo:**

Examinar a eficácia da acupressão aplicada no ponto P6 na redução de náuseas e vômitos durante a gravidez e sua influência no bem-estar psicossocial.

**Método:**

Estudo quase-experimental do tipo pré-teste e pós-teste, incluindo 61 gestantes (31 no grupo intervenção e 30 no grupo controle). Os dados foram coletados por meio do Formulário de Informações Descritivas da Gravidez, do Teste de Quantificação Única de Êmese na Gravidez (PUQE) e da Escala de Avaliação da Saúde Psicossocial na Gravidez (PPHAS).

**Resultados:**

Após a intervenção, o grupo que utilizou a pulseira de acupressão demonstrou uma redução significativamente maior nos escores PUQE em comparação ao grupo placebo. A análise de medidas repetidas revelou uma diminuição significativa e progressiva na gravidade das náuseas e vômitos no grupo da pulseira de acupressão do início até o quinto dia, enquanto nenhuma mudança significativa foi observada no grupo placebo. Os escores da PPHAS aumentaram significativamente em ambos os grupos após a intervenção; no entanto, não foi encontrada diferença estatisticamente significativa entre os grupos nos escores pós-intervenção.

**Conclusão:**

A acupressão no ponto P6 parece ser um método seguro, prático e não farmacológico que reduz eficazmente as náuseas e vômitos, podendo apoiar o bem-estar psicossocial no início da gravidez.

## INTRODUCTION

Pregnancy involves profound physiological and psychological changes. Among the most common challenges during this period are nausea and vomiting, which are typically most pronounced in the first trimester. These symptoms affect approximately 50–80% of pregnant women and can substantially compromise quality of life^(1)^. In cases of severe nausea and vomiting, the condition is classified as hyperemesis gravidarum. Hyperemesis gravidarum occurs in roughly 0.3–2% of pregnancies and is characterized by dehydration, electrolyte imbalances, malnutrition, and significant weight loss resulting from excessive nausea and vomiting^(2)^. If left untreated, it may lead to renal and hepatic dysfunction, vitamin deficiencies, and negative mental health outcomes for the mother. For the fetus, hyperemesis gravidarum is associated with an increased risk of intrauterine growth restriction, low birth weight, preterm birth, and perinatal mortality^(3)^. Therefore, early diagnosis and effective management of this condition are critical for the health of both mother and child.

Management of nausea and vomiting during pregnancy involves a combination of pharmacological and non- pharmacological approaches. Antiemetic medications are commonly used to manage nausea and vomiting; however, their use in pregnant women is often limited by side effects, including sedation, dizziness, and potential teratogenicity^(4)^. Consequently, interest in non-pharmacological interventions has increased in recent years. Among these approaches, dietary modifications, aromatherapy, relaxation techniques, herbal remedies, and acupressure are frequently adopted^(5)^.

Acupressure, a technique derived from traditional Chinese medicine, seeks to regulate the flow of energy by applying pressure to specific points on the body. Emerging evidence suggests that stimulation of the P6 (Neiguan) point, particularly on the wrist, can alleviate nausea and vomiting^(6,7)^. Owing to its safety, non-invasiveness, and ease of use in pregnant women, acupressure is increasingly regarded as a viable alternative or adjunct to pharmacological therapy^(8)^.

Hyperemesis gravidarum can lead to serious complications and may pose life-threatening risks for both mother and fetus. Therefore, safe and non-invasive interventions are particularly important, given the potential adverse effects of pharmacological treatments. In this regard, acupressure emerges as a promising non-pharmacological approach and has been shown to be effective not only for pregnancy-related nausea and vomiting but also for nausea and vomiting associated with surgery or chemotherapy^(9)^. Health professionals, particularly nurses, play a key role in educating, implementing, and monitoring interventions in pregnant women. Nurses assess the severity and characteristics of nausea and vomiting, develop individualized care plans, and monitor possible side effects of pharmacological treatments. In addition, they guide pregnant women in the use of non-pharmacological methods such as acupressure^(10)^. Teaching acupressure techniques, supervising their correct application, and evaluating their outcomes constitute an important component of holistic nursing care. Thus, a holistic approach to the care of pregnant women can be achieved.

There is a growing body of randomized controlled trials and systematic reviews evaluating the effectiveness of acupressure in the management of nausea and vomiting during pregnancy. In particular, studies have reported that acupressure applied to the P6 (Neiguan) point is effective in reducing these symptoms^(7,8, 11–13)^. However, several methodological limitations are evident in the existing literature. For instance, in some randomized controlled trials conducted in Türkiye, the absence of a placebo intervention in the control groups, compared to the acupressure wristband applied to the experimental groups, limits the interpretability of the findings^(11,13)^. Moreover, most of these studies have primarily focused on nausea and vomiting symptoms, without comprehensively addressing psychosocial well-being, which represents a critical dimension of the pregnancy experience. In addition, the clinical use and acceptability of acupressure may vary depending on cultural and healthcare system contexts, and evidence regarding its use during pregnancy in Türkiye remains limited. These gaps highlight the need for methodologically rigorous and context-specific research. Therefore, the present study aims to evaluate the effectiveness of acupressure on nausea and vomiting during pregnancy using a placebo-controlled design, as well as to examine its effects on psychosocial well-being. The findings are expected to contribute culturally sensitive and evidence-based insights to nursing practice.

H1: The use of acupressure wristbands is effective in reducing the frequency and severity of nausea and vomiting during pregnancy.
H2: Acupressure wristbands are more effective than placebo wristbands in reducing the frequency and severity of nausea and vomiting during pregnancy.
H3: The use of acupressure wristbands contributes to an improvement in the psychosocial well-being of pregnant women.

## METHOD

### Study Design

This research was conducted using a pre-test–post-test quasi-experimental design.

### Population

The study population consisted of all pregnant women experiencing nausea who presented to the pregnancy follow- up outpatient clinic of the Zeynep Kamil Women and Children Diseases Traning and Research Hospital between June 2021 and January 2022.

### Selection Criteria

The study used convenience sampling, a type of non-probability sampling method. The study sample comprised pregnant women who attended the pregnancy follow-up outpatient clinic of the same hospital during the specified period, had a gestational age of ≤16 weeks, and were identified as experiencing moderate to severe nausea and vomiting (PUQE score >7) according to the Pregnancy-Unique Quantification of Emesis (PUQE) Test.

### Sample Definition

The required sample size was determined using G*Power 3.1.9.2 software, based on a 95% confidence level, 80% statistical power (1–β), and a medium effect size (d = 0.50). According to these calculations, a minimum of 27 participants was required in each group. After assessing the inclusion criteria, pregnant women were assigned to the intervention and control groups using a simple randomization method. A random number generator (http://random.org) was used to allocate participants to the groups (intervention and control). Considering potential attrition, a larger number of participants was initially recruited.

At the beginning of the study, 70 pregnant women who met the inclusion criteria were enrolled, with 35 assigned to the experimental group and 35 to the control group. During the study period, four participants from the experimental group and five from the control group withdrew for various reasons. In the experimental group, withdrawals occurred due to difficulty adhering to the study protocol, loss of contact for personal reasons, and the emergence of pregnancy-related complications. In the control group, participants discontinued participation either because they perceived the placebo wristband as ineffective or developed pregnancy-related complications. Consequently, the analyses were performed on 61 pregnant women who completed the study, including 31 in the intervention group and 30 in the control group.

### Data Collection

After participants were informed about the purpose and process of the study, those who provided both verbal and written consent were assigned to either the experimental group (n = 31) and the control group (n = 30) according to a pre- prepared randomization list. Participants in the experimental group were given acupressure wristbands designed for nausea, while those in the control group received placebo wristbands. Both groups were provided with education on general strategies for managing nausea and vomiting during pregnancy. The educational content included recommendations such as consuming small and frequent meals, avoiding fatty or spicy foods, eating light snacks (e.g., salty crackers) before getting out of bed to reduce morning sickness, maintaining adequate hydration, ensuring sufficient rest, and using stress management techniques. In addition, information was provided about the physiological mechanisms of nausea and vomiting in pregnancy, and the importance of non-pharmacological interventions in symptom management was emphasized.

**Figure 1:**
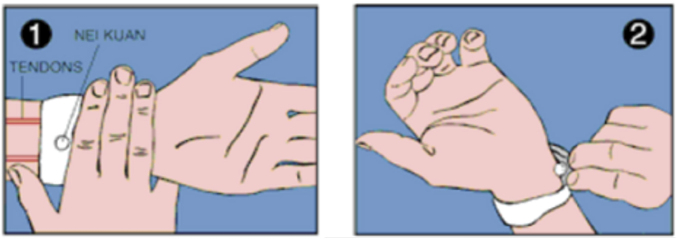
Identification of the p6 (neiguan) acupressure point and placement of the wristband.

**Figure 2:**
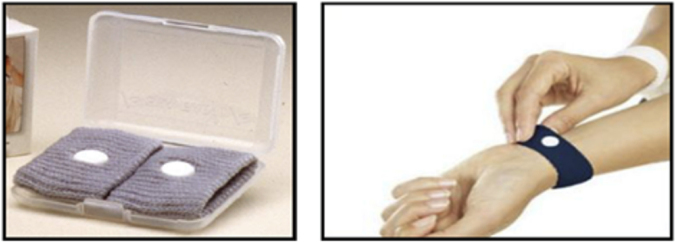
Application of the acupressure wristband.

Prior to the intervention, participants in the intervention group received both verbal and practical instruction on the correct use of the acupressure wristband. They were informed that the P6 (Neiguan) acupressure point is located on the inner wrist, approximately three finger-widths above the wrist crease, and that the white button on the wristband should be positioned directly over this point (Figures 1, 2). Participants were guided to locate the P6 point themselves using their own three-finger measurement and to place the wristband accordingly. The researcher then checked and confirmed the correct placement of the wristband for each participant.

Participants in the intervention group were asked to wear the acupressure wristband continuously on both wrists for five days. They were reminded that they could only temporarily remove the wristbands during sleep and while washing their hands or showering, but that they should put them back on as soon as possible to ensure continuous and effective pressure. The continuous use approach was chosen to enhance the therapeutic effect by providing uninterrupted stimulation to the P6 (Neiguan) point. Participants in the control group were given placebo wristbands that were identical in appearance and material to those used in the intervention group, but did not contain any buttons or stimulating components to create pressure on the acupressure point.

Participants were asked to wear the placebo wristbands continuously on both wrists for five days, following the same usage protocol as the intervention group. This approach was used to allow for the evaluation of differences between the groups solely related to the acupressure effect and to control for any potential placebo effect.

Literature reports that the duration and frequency of acupressure applications vary, with some studies reporting that wristbands were used for a week but at specific intervals throughout the day (e.g., three times a day)^(11,13)^. In this study, however, the application protocol was planned as continuous use for five days. This choice was made to increase participant compliance, reduce application errors, and ensure standardization by eliminating variability related to the frequency of daily use. Furthermore, considering that nausea and vomiting symptoms in early pregnancy can fluctuate daily, it was foreseen that the effect of a short but uninterrupted intervention could be evaluated more clearly. Accordingly, the intervention period was set at five days, and an evaluation was performed by a healthcare professional on the sixth day. This approach allowed for both the evaluation of the short-term effectiveness of the intervention and ensured the continuity of participant follow-up.

Data for the study were collected using the Pregnancy Descriptive Information Form, the Pregnancy-Unique Quantification of Emesis (PUQE) Test, and the Pregnancy Psychosocial Health Assessment Scale (PPHAS).

*Pregnancy Descriptive Information Form:* This form was developed by the researchers to collect data on the socio- demographic characteristics, health background, and obstetric history of the participants. It consisted of 17 items in total^(5,14,15)^.

*Pregnancy Pregnancy-Unique Quantification of Emesis (PUQE) Index:* The PUQE was originally developed by Rhodes et al. (1984) to evaluate chemotherapy-induced nausea and vomiting and was later adapted to measure the severity of nausea and vomiting during pregnancy^(16)^ Drawing on the Rhodes scoring system, the PUQE includes three items assessing the duration of nausea, the number of vomiting episodes, and the number of retching episodes. The total score ranges from 3 to 15, with values of 3–6 indicating mild, 7–12 moderate, and 13–15 severe nausea and vomiting^(14,16,17)^. Previous studies have shown a strong correlation between the PUQE and the original Rhodes Index. Furthermore, Sucu et al. (2009) reported that, in determining the need for hospitalization, the PUQE demonstrated a sensitivity of 94.74%, a specificity of 85.71%, a positive predictive value of 72%, and a negative predictive value of 97.67% when compared with physical examination^(14)^. These findings confirm that PUQE is a valid and reliable instrument for assessing the severity of nausea and vomiting during pregnancy.

*Pregnancy Psychosocial Health Assessment Scale (PPHAS):* The PPHAS, developed by Yıldız (2011), is a validated and reliable instrument designed to assess psychosocial well-being during pregnancy^(15)^. The scale consists of 46 items rated on a 5-point Likert-type format and includes six subscales: Pregnancy and Spousal Relationship (13 items), Anxiety and Stress (8 items), Domestic Violence (8 items), Need for Psychosocial Support (7 items), Familial Characteristics (4 items), and Physical- Psychosocial Changes Related to Pregnancy (6 items). Total scores range from 46 to 230. The mean score, obtained by dividing the total score by the number of items, ranges between 1 and 5, with values of 1.00–1.79 indicating “very poor,” 1.80–2.59 “poor,” 2.60–3.39 “moderate,” 3.40–4.19 “good,” and 4.20–5.00 “very good” psychosocial health. In the original study, the scale demonstrated a Cronbach’s alpha coefficient of 0.93,12 while in the present study, internal consistency remained high with a Cronbach’s alpha of 0.91.

During the initial interview, participants completed the Pregnancy Descriptive Information Form, the PUQE, and the PPHAS. Following completion of the questionnaires, participants received individualized instruction on strategies for managing nausea and were trained in the correct use of the wristbands.

Participants were instructed to wear the wristbands at home for five consecutive days and to complete the PUQE each evening at 10:00 pm. On the fifth day, they were also asked to complete the PPHAS once again. Throughout the home-based phase, the researchers provided regular online communication, including information, reminders, and consultation support.

On the fifth day of the intervention, online interviews lasting approximately 10–15 minutes were conducted with the participants. During these sessions, participants shared the results of the evaluation forms they had completed at home, and the data were recorded in individual case follow-up forms. In addition, participants’ opinions, suggestions, and questions regarding the intervention were gathered to provide complementary insights into its perceived effectiveness.

### Data Analysis and Treatment

Data was analyzed using IBM SPSS Statistics version 25.0. Descriptive statistics were first used to summarize the participants’ socio-demographic and obstetric characteristics. The chi-square (*χ^2^*) test was applied to examine whether the intervention and control groups were comparable in these characteristics. The normality of data distribution was evaluated using the Kolmogorov–Smirnov and Shapiro–Wilk tests, both of which indicated that the data followed a normal distribution. Between-group differences in mean scores were assessed using independent samples t-tests, while within- group changes over time were analyzed through repeated measures ANOVA. When the repeated measures analysis revealed a significant effect, post-hoc pairwise comparisons were performed using Bonferroni correction to identify the specific time points at which the differences occurred. A p-value of less than 0.05 was considered statistically significant in all analyses.

### Ethical Aspects

Ethical approval for the study was obtained from the Zeynep Kamil Women and Children Diseases Traning and Research Hospital Ethics Committee (dated 06.05.2020, No. 86). Necessary institutional permissions were also granted by the healthcare facilities where the research was conducted. All participants were informed verbally and in writing about the study’s purpose, procedures, and their right as participants. Written informed consent was obtained from each participant prior to inclusion in the study.

### Limitations

Although the sample size in this study is sufficient according to the power analysis results, the generalizability of the findings is limited due to its relatively small size. Therefore, further studies with larger and different samples are recommended.

## RESULTS

The acupressure wristband and placebo wristband groups were comparable in terms of sociodemographic characteristics, with no statistically significant differences between the groups (p > 0.05) (Table 1).

**Table 1:** Distribution of participants by demographic and health characteristics – Istanbul, Türkiye, 2021-2022.

Characteristics	Acupressure wristband (n:31)		Placebo wristband (n:30)		*χ^2^*	p*
	n	%	n	%		
Age (years) mean ± sd (min-max)	27.4 ± 5.8 (20–39)		28.3 ± 5.5 (21–42)		17.688	0.409
Education status						
Primary school	3	9.7	4	13.3	7.726	0.102
Secondary school	11	35.5	4	13.3		
High school	9	29.0	11	36.7		
Bachelor’s	8	25.8	11	36.7		
Employment status						
Employed	7	22.6	10	33.3	0.877	0.258
Unemployed	24	77.4	20	66.7		
Social security coverage						
Yes	28	90.3	29	96.7	1.001	0.319
No	3	9.7	1	3.3		
Family structure						
Nuclear	28	90.3	30	100.0	3.53	0.125
Extended	3	9.7	0	0.0		
Chronic illness						
Yes	9	2.0	7	23.3	0.256	0.416
No	22	71.0	23	76.7		
Smoking habits						
Yes	5	16.1	6	20.0	1.581	0.454
No	26	83.9	24	80.0		

*χ^2^* chi square test

*p < 0.05.

The acupressure wristband and placebo wristband groups were similar in terms of obstetric characteristics, with no statistically significant differences between groups (p > 0.05) (Table 2).

**Table 2:** Distribution of participants by obstetric characteristics – Istanbul, Türkiye, 2021-2022.

Obstetric characteristics	Acupressure wristband (n:31)		Placebo wristband (n:30)		*χ^2^*	p*
	n	%	n	%		
Gravida Mean ± SD (min-max)	2.1 ± 1.42 (1–7)		2.6 ± 1.37 (1–6)		6.045	0.355
Gestational week Mean ± SD (min-max)	13.3 ± 1.42 (8–15)		12.8 ± 1.42 (7–15)		4.442	0.265
Number of living children						
0	17	54.8	14	46.7	1.994	0.737
1	9	29.0	10	33.3		
2	3	8.7	4	13.3		
3	2	6.5	2	6.7		
Parity						
Nulliparous	12	38.7	11	36.7	0.075	0.524
Multiparous	19	61.3	19	63.3		
Pregnancy intention status						
Intended	25	80.6	25	83.3	3.985	0.245
Unintended	6	19.4	5	16.7		
History of nausea and vomiting in previous pregnancy						
Yes	14	45.2	13	43.3	2.608	0.271
No	5	16.1	6	20.0		
First pregnancy	12	38.7	11	36.7		
Family history of nausea and vomiting					1.761	0.164
Yes	24	77.4	27	90.0		
No	7	22.6	3	10.0		
Knowledge about pregnancy, childbirth, and the postpartum period						
Adequate	15	48.4	20	66.7	2.069	0.118
Inadequate	16	51.6	10	33.3		
Participation in antenatal education programs						
Yes	6	19.4	8	26.7	4.546	0.135
No	25	80.6	22	73.3		
Regular antenatal care						
Yes	29	93.5	27	90.0	1.816	0.172
No	2	6.4	3	10.0		

*χ^2^* chi square test

*p < 0.05.

Table 3 presents the comparison of PUQE scores between the acupressure wristband and placebo wristband groups. Prior to the intervention, the mean PUQE score was 9.65 ± 2.35 in the acupressure wristband group and 9.62 ± 2.20 in the placebo wristband group, with no statistically significant difference observed (p = 0.059). Following the intervention, the mean score in the acupressure wristband group decreased to 5.26 ± 2.14, while the placebo wristband group had a mean score of 7.86 ± 2.37. This difference between the groups was statistically significant (p = 0.003).

**Table 3:** Comparison of intragroup PUQE scores in pregnant women – Istanbul, Türkiye, 2021-2022.

Groups	X ± SD	p
Pre-Intervention		
Acupressure Wristband	9.65 ± 2.35	0.059
Placebo Wristband	9.62 ± 2.20	
Post-Intervention		
Acupressure Wristband	5.26 ± 2.14	0.003*
Placebo Wristband	7.86 ± 2.37	

Independent Sample t test

*p < 0.05.

Table 4 presents the distribution of nausea and vomiting severity (PUQE scores) in pregnant women over the course of the intervention. In the acupressure wristband group, the mean PUQE score was 9.65 ± 2.35 at baseline and decreased from the very first day: 7.93 ± 1.50 on day one, 5.80 ± 1.77 on day two, 5.67 ± 2.44 on day three, 5.34 ± 2.18 on day four, and 5.26 ± 2.14 on day five. Repeated measures analysis confirmed that this reduction was statistically significant (p < 0.001). Post-hoc comparisons indicated that the most significant differences occurred between the baseline measurement and days two through five. In contrast, the placebo wristband group showed only a slight downward trend in the mean PUQE scores over the five days (9.62 ± 2.20 at baseline; 7.86 ± 2.37 on day five), which was not statistically significant (p > 0.05). These results suggest that acupressure wristband application effectively reduces the severity of nausea and vomiting in pregnant women, while the placebo wristband does not produce a comparable effect.

**Table 4:** Comparison of PUQE scores between experimental and control groups – Istanbul, Türkiye, 2021-2022.

Groups	Intervention process	X ± SD	p*
Acupressure Wristband	Initial	9.65 ± 2.35	0.000*
	Day 1	7.93 ± 1.50	
	Day 2	5.80 ± 1.77	
	Day 3	5.67 ± 2.44	
	Day 4	5.34 ± 2.18	
	Day 5	5.26 ± 2.14	
Placebo Wristband	Initial	9.62 ± 2.20	0.063
	Day 1	9.36 ± 2.20	
	Day 2	9.10 ± 1.90	
	Day 3	8.46 ± 2.11	
	Day 4	8.10 ± 1.98	
	Day 5	7.86 ± 2.37	

ANOVA test

*p < 0.05.

Table 5 presents the comparison of PPHAS scores between the acupressure wristband and placebo wristband groups throughout the intervention. Prior to the intervention, the mean PPHAS score was 2.94 ± 0.27 in the acupressure wristband group and 2.99 ± 0.31 in the placebo group, with no statistically significant difference between the groups (p = 0.746). After the intervention, the mean scores increased to 3.22 ± 0.24 in the acupressure wristband group and 3.16 ± 0.29 in the placebo wristband group, yet this difference remained statistically non-significant (p = 0.355). Intragroup comparisons, however, revealed a significant improvement in both groups from pre- to post-intervention (acupressure group, p = 0.000; placebo group, p = 0.000). These findings suggest that while both wristband interventions were associated with enhanced psychosocial well- being in pregnant women, the addition of acupressure did not produce a statistically greater effect compared to the placebo.

**Table 5:** Comparison of PPHAS scores – Istanbul, Türkiye, 2021-2022.

Groups		X ± SD	p*
Pre-Intervention	Acupressure Wristband	2.94 ± 0.27	0.746
	Placebo Wristband	2.99 ± 0.31	
Post-Intervention	Acupressure Wristband	3.22 ± 0.24	0.355
	Placebo Wristband	3.16 ± 0.29	
Acupressure Wristband	Pre-Intervention	2.94 ± 0.27	0.000*
	Post-Intervention	3.22 ± 0.24	
Placebo Wristband	Pre-Intervention	2.99 ± 0.31	0.000*
	Post-Intervention	3.16 ± 0.29	

PPHAS: Pregnancy Psychosocial Health Assessment Scale, Independent Sample t test, ANOVA test

*p < 0.05.

## DISCUSSION

This study examined the effectiveness of acupressure applied to the P6 point in reducing nausea and vomiting during pregnancy, as well as its potential impact on psychosocial health. Pregnancy-related nausea and vomiting are often linked to elevated levels of beta-human chorionic gonadotropin (β-hCG). Given the possible risks of pharmacological treatments to the fetus, non-pharmacological approaches are increasingly favored for symptom management^(11)^. Acupressure represents one such safe and non-invasive method. Evidence from the literature indicates that stimulation of specific acupressure points may increase β-endorphin levels, and that higher concentrations of endorphins and 5-hydroxytryptamine (5-HT) may help alleviate nausea and vomiting symptoms^(7)^. Studies investigating the effectiveness of acupressure for managing nausea and vomiting during pregnancy vary in terms of comparison groups, intervention duration, and follow-up periods. One study, for example, compared acupressure, placebo, and control groups. In a study conducted in Iran, the experimental group received acupressure at the P6 point using the Sea-Band button for three days, the placebo group wore the Sea-Band without applying pressure to the P6 point, and the control group received only dietary recommendations, similar to those given to the other two groups. The frequency and severity of nausea and vomiting were assessed twice daily for six days, starting from the fourth day of the intervention. After three days, both the acupressure and placebo groups demonstrated a significant reduction in the frequency, duration, and severity of nausea, as well as in the frequency of vomiting, while no such reduction was observed in the control group^(12)^. In another study, no treatment was administered to any group during the first three days; subsequently, acupressure at the P6 point was applied to the experimental group for a period of four days. Although no significant differences were observed between the acupressure and placebo groups regarding nausea and vomiting, both groups experienced significantly lower symptom levels compared to the control group^(18)^. In these studies, the severity of nausea and vomiting did not vary between participants receiving true acupressure and those receiving placebo, suggesting that, beyond the physiological effects of acupressure, the patients’ belief in the intervention and the act of tactile stimulation itself may produce a meaningful therapeutic effect. In another type of study, the effectiveness of acupressure was compared with standard treatment methods. In a study conducted in Malaysia, the experimental group received acupressure at the P6 point three times daily for at least 10 minutes, in addition to intravenous fluids and the rescue antiemetic metoclopramide (10 mg). The control group received intravenous fluids and regular metoclopramide (10 mg) every 8 hours for 24 hours. The findings indicated that the experimental group experienced reduced nausea and vomiting, and their need for additional antiemetics was significantly lower than that of the control group. The study demonstrates that, when applied alongside standard care, acupressure has a significant effect in reducing nausea and vomiting^(8)^. In another study, the effectiveness of acupressure was compared across acupressure, placebo, and standard treatment groups. Conducted in Iran, the study assigned one group to receive P6 acupressure four times daily for 10 minutes, a second group to receive sham (placebo) acupressure, and a third group to receive vitamin B6 and metoclopramide. Assessments conducted before the intervention, as well as on the first and fifth days post-intervention, revealed that by the fifth day, significant differences emerged in vomiting frequency, gagging distress, vomiting distress, nausea duration, nausea distress, vomiting volume, nausea frequency, and retching frequency. These findings suggest that stimulation of the P6 acupressure point can effectively reduce the severity of nausea, vomiting, and retching in pregnant women^(13)^. In a separate set of studies, outcomes were compared between acupressure and control groups. In Türkiye, one study assigned the experimental group to receive P6 acupressure three times daily for one week, while the control group received no intervention. The results indicated a significant reduction in nausea and vomiting in the acupressure group^(13,19)^. Similarly, in another study, the experimental group received acupressure for one week, while the control group did not receive any method for relieving nausea and vomiting; once again, acupressure significantly reduced nausea and vomiting scores, while no notable changes were observed in the control group^(11)^. However, the results of these two studies conducted in Türkiye reveal some variability regarding the effectiveness of acupressure. Another study type focused on comparing acupressure with placebo. In one such study, the experimental group received P6 acupressure for approximately 60 minutes daily over seven consecutive days, while the control group received only P6 contact, following the same procedure and duration, without active pressure. In the experimental group, both the severity and frequency of nausea and vomiting were significantly reduced following the initiation of treatment compared to the placebo group^(20)^. The study concluded that acupressure at the P6 point was more effective in alleviating nausea and vomiting than the placebo intervention. Evidence from the literature further supports these findings. Two meta- analyses have evaluated the effectiveness of acupressure for pregnancy-related nausea and vomiting. In one meta-analysis, which included 11 randomized controlled trials with a total of 1,378 pregnant women, acupressure was associated with greater improvements in nausea and vomiting symptoms compared to both placebo and control groups. In addition, acupressure demonstrated benefits in preventing nausea and vomiting, reducing the length of hospital stay, and enhancing patient satisfaction relative to placebo^(6)^. Another meta-analysis, including 33 studies with 3,390 pregnant women, similarly found that acupressure significantly reduced nausea scores, shortened hospital stay, and improved overall quality of life^(7)^.

The findings of this study are consistent with those reported in the literature, indicating that stimulation of the P6 acupressure point may effectively alleviate nausea and vomiting during pregnancy. However, some studies have found no significant differences between acupressure and sham (placebo) interventions, suggesting that the benefits of acupressure may arise not only from physiological mechanisms but also from psychological or placebo effects. Differences in study design, duration and frequency of the intervention, timing of outcome assessments, and cultural context may explain inconsistencies across studies. Taken together, meta-analytic evidence generally supports the use of acupressure as a complementary approach for managing pregnancy-related nausea and vomiting; however, these findings highlight the need for methodological standardization and more rigorous randomized controlled trials directly comparing acupressure with sham (placebo) interventions.

Pregnant women experiencing nausea and vomiting often report elevated levels of stress, anxiety, depression, and concerns about potential harm to their infants^(21,22)^. However, studies examining the effects of acupressure on psychosocial well-being in this population are limited. In a study, acupressure was applied to the P6 point to relieve nausea and vomiting and to the L14 point to reduce anxiety, for 15 minutes each, daily for seven consecutive mornings upon waking. Both intervention groups received the same acupressure protocol, while the control group received no intervention. The study found that acupressure at the P6 and L14 points effectively reduced anxiety^(23)^. In the present study, psychosocial well-being improved in both the acupressure and control groups; however, no statistically significant difference between the groups was observed. These findings suggest that the impact of acupressure on psychosocial health may be influenced by factors such as individual variability, the duration and location of application, timing of assessment, or placebo effects. Further research is needed to clarify the role of acupressure in enhancing psychosocial well-being among pregnant women.

The study was conducted at a single center with a relatively small sample size, which restricts the generalizability of the findings. The long-term effects of the intervention were not assessed. Furthermore, the increase in psychosocial well-being observed in the control group suggests that placebo effects could not be entirely excluded. Future research should aim to confirm these findings through larger, multicenter, and long-term randomized controlled trials.

## CONCLUSION

This study assessed the effect of acupressure applied to the P6 point on the severity of nausea and vomiting and on psychosocial well-being in pregnant women. The findings indicate that acupressure is effective in alleviating nausea and vomiting symptoms during pregnancy. Although improvements in psychosocial health were observed following acupressure application, these changes were not statistically significant compared to the control group. These results suggest that acupressure may contribute to psychosocial well-being in addition to providing physical relief, but the effect appears to be limited. Given its safety, non-invasiveness, low cost, and ease of application, acupressure can be considered a practical method for managing pregnancy-related nausea and vomiting. Incorporating acupressure into a holistic nursing care approach may support both symptom management and psychosocial needs in pregnant women. Further large-scale, high-quality clinical trials are necessary to confirm its efficacy and long-term benefits.

## Data Availability

The entire dataset supporting the results of this study is available upon request to the corresponding author.

## References

[1] American College of Obstetricians and Gynecologists (2018). ACOG practice bulletin no. 189: nausea and vomiting of pregnancy.. Obstet Gynecol.

[2] Fejzo MS, MacGibbon KW (2012). Hyperemesis gravidarum: it is time to put an end to the misguided theory of a psychiatric etiology.. Gen Hosp Psychiatry.

[3] Verberg MF, Gillott DJ, Al-Fardan N, Grudzinskas JG (2005). Hyperemesis gravidarum, a literature review.. Hum Reprod Update.

[4] Matthews A, Haas DM, O’Mathúna DP, Dowswell T (2015). Interventions for nausea and vomiting in early pregnancy.. Cochrane Database Syst Rev.

[5] Smith C, Crowther C, Beilby J (2016). Acupuncture to treat nausea and vomiting in early pregnancy: a randomized controlled trial.. Birth.

[6] Wang X, Yang G, Li K, Yang F, Liang X, Wu S (2024). Efficacy and safety of acupressure in nausea and vomiting during pregnancy: a systematic review and meta-analysis of randomized controlled trials.. Arch Gynecol Obstet.

[7] Gong J, Gu D, Wang H, Zhang F, Shen W, Yan H (2024). Effect of acupressure in nausea and vomiting treatment during pregnancy: a meta-analysis.. Explore (NY).

[8] Mohd Nafiah NA, Chieng WK, Zainuddin AA, Chew KT, Kalok A, Abu MA (2022). Effect of acupressure at P6 on nausea and vomiting in women with hyperemesis gravidarum: a randomized controlled trial.. Int J Environ Res Public Health.

[9] Einarson TR, Piwko C, Koren G (2013). Quantifying the global rates of nausea and vomiting of pregnancy: a meta-analysis.. J Popul Ther Clin Pharmacol.

[10] Topçu GB, Aydın Ateş N, Küğcümen G (2019). Gebelikte bulantı ve kusma yönetimi.. J Cumhuriyet Univ Health Sci Inst..

[11] Yılmaz MP, Yazıcı S, Yılmaz I (2023). Effect of acupressure at PC6 on nausea and vomiting during pregnancy: a randomized controlled trial.. J Acupunct Meridian Stud.

[12] Mobarakabadi SS, Shahbazzadegan S, Ozgoli G (2020). The effect of P6 acupressure on nausea and vomiting of pregnancy: a randomized, single-blind, placebo-controlled trial.. Adv Integr Med.

[13] Şolt Kırca A, Gül DK (2020). Effects of acupressure applied to P6 point on nausea vomiting in pregnancy: a double-blind randomized controlled trial.. Altern Ther Health Med.

[14] Sucu M, Büyükkurt S, Evrüke İC, Demir SC, Özgünen FT, Kadayıfçı O (2009). Gebelikte bulantı kusması olan hastaların hastaneye yatış endikasyonlarının değerlendirilmesinde PUQE testinin yeri.. Turk Klin J Gynecol Obstet..

[15] Yıldız H (2011). Gebelikte psikososyal sağlığı değerlendirme ölçeği geliştirme çalışması.. Maltepe Univ Hemşirelik Bilim Sanatı Derg..

[16] Koren G, Piwko C, Ahn E, Boskovic R, Maltepe C, Einarson A, Navioz Y (2005). Validation studies of the Pregnancy Unique-Quantification of Emesis.. J Obstet Gynaecol..

[17] Köken G, Coşar E, Şahin FK, Arıöz DT, Yeşildağer E, Yılmazer M (2009). Erken gebelikte bulantı ve kusmaya etki eden faktörler.. TJOD Derg.

[18] Saberi F, Sadat Z, Abedzadeh-Kalahroudi M, Taebi M (2012). Impact of acupressure on nausea and vomiting during pregnancy.. Feyz Med Sci J..

[19] Tara F, Bahrami-Taghanaki H, Amini Ghalandarabad M, Zand-Kargar Z, Azizi H, Esmaily H (2020). The effect of acupressure on the severity of nausea, vomiting, and retching in pregnant women: a randomized controlled trial.. Complement Med Res.

[20] Safaa GA, Ayat MAE (2019). Effect of acupressure on nausea and vomiting during pregnancy.. Med J Cairo Univ.

[21] Kramer J, Bowen A, Stewart N, Muhajarine N (2013). Nausea and vomiting in pregnancy: prevalence, severity, and relation to psychosocial health.. MCN Am J Matern Child Nurs.

[22] Beyazit F, Sahin B (2018). Effect of nausea and vomiting on anxiety and depression levels in early pregnancy.. Eurasian J Med.

[23] Mudlikah S (2023). Acupressure technique point P6 (Nei Guan) to reduce nausea and vomiting and point L14 (Hegu) to reduce anxiety in pregnant women.. EMBRIO J Kebidanan..

