# Supplementary material for: Effectiveness of acupressure in managing nausea, vomiting, and psychosocial well-being during pregnancy
Source: Rev Esc Enferm USP. 2026 Jul 24;60:e20260082. doi: 10.1590/1980-220X-REEUSP-2026-0082en (PMC13404301; doi:10.1590/1980-220X-REEUSP-2026-0082en)
Supplement: Translation

# Translation: Eficacia de la acupresión en el tratamiento de las náuseas, los vómitos y el bienestar psicosocial durante el embarazo

**DOI:** 10.1590/1980-220X-REEUSP-2026-0082es

**Authors:** Yeliz Doğan Merih, Zehra Acar, Özlem Karabulut, Mehlika Aslan

**Affiliations:** 1 University of Health Sciences, Hamidiye Faculty of Nursing, Istanbul, Türkiye.; 2 Independent Researcher, Istanbul, Türkiye.; 3 Istanbul Univesity, Faculty of Nursing, Istanbul, Türkiye.

**Keywords:** Acupresión, Eficacia, Náusea, Embarazo, Vómito, Muñeca

## Abstract

**Objetivo:**

Evaluar la eficacia de la acupresión aplicada en el punto P6 para reducir las náuseas y los vómitos durante el embarazo y su influencia en el bienestar psicosocial.

**Método:**

Este estudio cuasi-experimental de pretest-post test incluyó a 61 mujeres embarazadas (31 en el grupo de intervención y 30 en el grupo de control). Los datos se recopilaron mediante el Formulario de Información Descriptiva del Embarazo, la Prueba de Cuantificación de la Emesis Específica del Embarazo (Pregnancy-Unique Quantification of Emesis-PUQE) y la Escala de Evaluación de la Salud Psicosocial durante el Embarazo (Pregnancy Psychosocial Health Assessment Scale – PPHAS).

**Resultados:**

Tras la intervención, el grupo de la pulsera de acupresión mostró una reducción significativamente mayor en las puntuaciones del PUQE en comparación con el grupo placebo. El análisis de medidas repetidas mostró una disminución significativa y progresiva en la gravedad de las náuseas y los vómitos en el grupo de la pulsera de acupresión desde el inicio hasta el quinto día, mientras que no se observó ningún cambio significativo en el grupo placebo. Las puntuaciones del PPHAS aumentaron significativamente en ambos grupos después de la intervención; sin embargo, no se encontró ninguna diferencia estadísticamente significativa entre los grupos en las puntuaciones posteriores a la intervención.

**Conclusión:**

La acupresión en P6 parece ser un método seguro, práctico y no farmacológico que reduce eficazmente las náuseas y los vómitos y puede favorecer el bienestar psicosocial en el inicio del embarazo.

## INTRODUCCIÓN

El embarazo conlleva profundos cambios fisiológicos y psicológicos. Entre los problemas más comunes durante este período se encuentran las náuseas y los vómitos, que suelen ser más pronunciados en el primer trimestre. Estos síntomas afectan aproximadamente al 50–80 % de las mujeres embarazadas y pueden comprometer sustancialmente la calidad de vida^(1)^. En casos de náuseas y vómitos intensos, la afección se clasifica como hiperémesis gravídica. La hiperémesis gravídica se presenta en aproximadamente el 0,3–2 % de los embarazos y se caracteriza por deshidratación, desequilibrios electrolíticos, desnutrición y pérdida de peso significativa como resultado de náuseas y vómitos excesivos^(2)^. Si no se trata, puede conducir a disfunción renal y hepática, deficiencias vitamínicas y consecuencias negativas para la salud mental de la madre. Para el feto, la hiperémesis gravídica se asocia con un mayor riesgo de restricción del crecimiento intrauterino, bajo peso al nacer, parto prematuro y mortalidad perinatal^(3)^. Por lo tanto, el diagnóstico temprano y el manejo eficaz de esta afección son fundamentales para la salud tanto de la madre como del niño.

El manejo de las náuseas y los vómitos durante el embarazo implica una combinación de enfoques farmacológicos y no farmacológicos. Los medicamentos antieméticos se utilizan comúnmente para controlar las náuseas y los vómitos; sin embargo, su uso en mujeres embarazadas suele verse limitado por los efectos secundarios, que incluyen sedación, mareos y posible teratogenicidad^(4)^. En consecuencia, el interés en las intervenciones no farmacológicas ha aumentado en los últimos años. Entre estos enfoques, se adoptan con frecuencia modificaciones dietéticas, aromaterapia, técnicas de relajación, remedios a base de hierbas y acupresión^(5)^.

La acupresión, una técnica derivada de la medicina tradicional china, busca regular el flujo de energía mediante la aplicación de presión en puntos específicos del cuerpo. Evidencias emergentes sugieren que la estimulación del punto P6 (Neiguan), particularmente en la muñeca, puede aliviar las náuseas y los vómitos^(6,7)^.

Debido a su seguridad, carácter no invasivo y facilidad de uso en mujeres embarazadas, la acupresión se considera cada vez más como una alternativa viable o un complemento a la terapia farmacológica^(8)^.

La hiperémesis gravídica puede provocar complicaciones graves y suponer riesgos que pongan en peligro la vida tanto de la madre como del feto. Por lo tanto, las intervenciones seguras y no invasivas son particularmente importantes, dados los posibles efectos adversos de los tratamientos farmacológicos. En este sentido, la acupresión surge como un enfoque no farmacológico prometedor y ha demostrado ser eficaz no solo para las náuseas y los vómitos relacionados con el embarazo, sino también para los náuseas y los vómitos asociados con la cirugía o la quimioterapia^(9)^. Los profesionales de la salud, en particular las enfermeras, desempeñan un papel clave en la educación, la implementación y el seguimiento de las intervenciones en mujeres embarazadas. Las enfermeras evalúan la gravedad y las características de las náuseas y los vómitos, desarrollan planes de atención individualizados y monitorean los posibles efectos secundarios de los tratamientos farmacológicos. Además, orientan a las mujeres embarazadas en el uso de métodos no farmacológicos, como la acupresión^(10)^. La enseñanza de técnicas de acupresión, la supervisión de su correcta aplicación y la evaluación de sus resultados constituyen un componente importante de la atención de enfermería holística. De este modo, se puede lograr un enfoque holístico en la atención a las mujeres embarazadas.

Existe un creciente conjunto de ensayos controlados aleatorios y revisiones sistemáticas que evalúan la eficacia de la acupresión en el manejo de las náuseas y los vómitos durante el embarazo. En particular, los estudios han informado que la acupresión aplicada en el punto P6 (Neiguan) es eficaz para reducir estos síntomas^(7,8,11–13)^. Sin embargo, son evidentes varias limitaciones metodológicas en la literatura existente. Por ejemplo, en algunos ensayos controlados aleatorios realizados en Turquía, la ausencia de una intervención con placebo en los grupos de control, en comparación con la pulsera de acupresión aplicada a los grupos experimentales, limita la interpretabilidad de los hallazgos^(11,13)^. Además, la mayoría de estos estudios se han centrado principalmente en los síntomas de náuseas y vómitos, sin abordar de manera integral el bienestar psicosocial, lo cual representa una dimensión crítica de la experiencia del embarazo. Además, el uso clínico y la aceptabilidad de la acupresión pueden variar según los contextos culturales y del sistema de salud, y la evidencia sobre su uso durante el embarazo en Turquía sigue siendo limitada. Estas brechas resaltan la necesidad de una investigación metodológicamente rigurosa y específica para cada contexto. Por lo tanto, el presente estudio tiene como objetivo evaluar la eficacia de la acupresión en las náuseas y los vómitos durante el embarazo utilizando un diseño controlado con placebo, así como examinar sus efectos sobre el bienestar psicosocial. Se espera que los hallazgos aporten conocimientos culturalmente sensibles y basados en la evidencia a la práctica de la enfermería.

H1: El uso de pulseras de acupresión es eficaz para reducir la frecuencia y la gravedad de las náuseas y los vómitos durante el embarazo.
H2: Las pulseras de acupresión son más eficaces que las pulseras de placebo para reducir la frecuencia y la gravedad de las náuseas y los vómitos durante el embarazo.
H3: El uso de pulseras de acupresión contribuye a una mejora en el bienestar psicosocial de las mujeres embarazadas.

## MÉTODO

### Diseño del Estudio

Esta investigación se llevó a cabo utilizando un diseño cuasi- experimental de pre-prueba y post-prueba.

### Población

La población del estudio estuvo compuesta por todas las mujeres embarazadas que experimentaban náuseas y que acudieron a la clínica ambulatoria de seguimiento del embarazo del Hospital de Formación e Investigación Zeynep Kamil para las Enfermedades de la Mujer y del Niño entre junio de 2021 y enero de 2022.

### Criterios de Selección

El estudio utilizó un muestreo por conveniencia, un tipo de método de muestreo no probabilístico. La muestra del estudio estuvo compuesta por mujeres embarazadas que acudieron a la clínica ambulatoria de seguimiento del embarazo del mismo hospital durante el período especificado, tenían una edad gestacional ≤16 semanas y se identificó que presentaban náuseas y vómitos de moderados a graves (puntuación PUQE >7) según la prueba de cuantificación de la emesis específica del embarazo (PUQE).

### Definición de la Muestra

El tamaño de muestra requerido se determinó utilizando el software G*Power 3,1,9,2, basándose en un nivel de confianza del 95 %, una potencia estadística del 80 % (1–β) y un tamaño del efecto medio (d = 0,50). Según estos cálculos, se requería un mínimo de 27 participantes en cada grupo. Tras evaluar los criterios de inclusión, se asignó a las mujeres embarazadas a los grupos de intervención y control mediante un método de aleatorización simple.

Se utilizó un generador de números aleatorios (http://random.org) para asignar a las participantes a los grupos (de intervención y de control). Teniendo en cuenta el posible desgaste, inicialmente se reclutó un mayor número de participantes.

Al inicio del estudio, se inscribieron 70 mujeres embarazadas que cumplían los criterios de inclusión, de las cuales 35 fueron asignadas al grupo experimental y 35 al grupo de control. Durante el período de estudio, cuatro participantes del grupo experimental y cinco del grupo de control se retiraron por diversas razones. En el grupo experimental, los retiros se debieron a dificultades para cumplir con el protocolo del estudio, pérdida de contacto por motivos personales y la aparición de complicaciones relacionadas con el embarazo. En el grupo de control, las participantes abandonaron la participación ya sea porque percibieron que la pulsera placebo era ineficaz o porque desarrollaron complicaciones relacionadas con el embarazo. En consecuencia, los análisis se realizaron con 61 mujeres embarazadas que completaron el estudio, incluidas 31 del grupo de intervención y 30 del grupo de control.

### Recolección de Datos

Después de que se informara a las participantes sobre el propósito y el proceso del estudio, aquellas que dieron su consentimiento tanto verbal como por escrito fueron asignadas al grupo experimental (n = 31) o al grupo de control (n = 30) de acuerdo con una lista de aleatorización preparada previamente. A las participantes del grupo experimental se les entregaron pulseras de acupresión diseñadas para las náuseas, mientras que las del grupo de control recibieron pulseras placebo. A ambos grupos se les proporcionó información sobre estrategias generales para el manejo de las náuseas y los vómitos durante el embarazo. El contenido educativo incluía recomendaciones tales como consumir comidas pequeñas y frecuentes, evitar alimentos grasos o picantes, comer bocadillos ligeros (p. ej., galletas saladas) antes de levantarse de la cama para reducir las náuseas matutinas, mantener una hidratación adecuada, garantizar un descanso suficiente y utilizar técnicas de manejo del estrés. Además, se proporcionó información sobre los mecanismos fisiológicos de las náuseas y los vómitos durante el embarazo, y se hizo hincapié en la importancia de las intervenciones no farmacológicas en el manejo de los síntomas.

Antes de la intervención, las participantes del grupo de intervención recibieron instrucciones tanto verbales como prácticas sobre el uso correcto de la pulsera de acupresión. Se les informó de que el punto de acupresión P6 (Neiguan) se encuentra en la parte interna de la muñeca, aproximadamente a tres dedos de distancia por encima del pliegue de la muñeca, y que el botón blanco de la pulsera debía colocarse directamente sobre este punto (Figuras 1, 2). Se indicó a los participantes que localizaran ellos mismos el punto P6 utilizando su propia medida de tres dedos y que colocaran la pulsera en consecuencia. A continuación, el investigador verificó y confirmó la colocación correcta de la pulsera en cada participante.

**Figura 1:** Identificación del punto de acupresión p6 (neiguan) y colocación de la pulsera.

**Figura 2:** Uso de la pulsera de acupresión.

Se pidió a los participantes del grupo de intervención que llevaran puesta la pulsera de acupresión de forma continua en ambas muñecas durante cinco días. Se les recordó que solo podían quitarse las pulseras temporalmente mientras dormían y mientras se lavaban las manos o se duchaban, pero que debían volver a ponérselas lo antes posible para garantizar una presión continua y eficaz. Se optó por el enfoque de uso continuo para potenciar el efecto terapéutico mediante la estimulación ininterrumpida del punto P6 (Neiguan). A los participantes del grupo de control se les entregaron pulseras placebo que eran idénticas en apariencia y material a las utilizadas en el grupo de intervención, pero que no contenían botones ni componentes estimulantes para ejercer presión sobre el punto de acupresión.

Se pidió a los participantes que usaran las pulseras placebo de manera continua en ambas muñecas durante cinco días, siguiendo el mismo protocolo de uso que el grupo de intervención. Este enfoque se utilizó para permitir la evaluación de las diferencias entre los grupos relacionadas únicamente con el efecto de la acupresión y para controlar cualquier posible efecto placebo.

La literatura indica que la duración y la frecuencia de las aplicaciones de acupresión varían; algunos estudios informan que las pulseras se utilizaron durante una semana, pero a intervalos específicos a lo largo del día (por ejemplo, tres veces al día)^(11,13)^. En este estudio, sin embargo, el protocolo de aplicación se planificó como un uso continuo durante cinco días. Esta decisión se tomó para aumentar el cumplimiento de las participantes, reducir los errores de aplicación y garantizar la estandarización al eliminar la variabilidad relacionada con la frecuencia de uso diario. Además, teniendo en cuenta que los síntomas de náuseas y vómitos en el embarazo temprano pueden fluctuar diariamente, se previó que el efecto de una intervención breve pero ininterrumpida podría evaluarse con mayor claridad. En consecuencia, el período de intervención se fijó en cinco días, y un profesional de la salud realizó una evaluación el sexto día. Este enfoque permitió tanto la evaluación de la eficacia a corto plazo de la intervención como la garantía de la continuidad del seguimiento de las participantes.

Los datos para el estudio se recolectaron utilizando el Formulario de Información Descriptiva del Embarazo, la Prueba de Cuantificación de la Emesis Específica del Embarazo (Pregnancy-Unique Quantification of Emesis – PUQE) y la Escala de Evaluación de la Salud Psicosocial del Embarazo (Pregnancy Psychosocial Health Assessment Scale-PPHAS).

*Formulario de Información Descriptiva del Embarazo:* Este formulario fue desarrollado por los investigadores para recolectar datos sobre las características sociodemográficas, los antecedentes de salud y el historial obstétrico de las participantes. Constaba de 17 ítems en total^(5,14,15)^.

*Índice de Cuantificación de la Emesis Específica del Embarazo (PUQE):* El PUQE fue desarrollado originalmente por Rhodes et al. (1984) para evaluar las náuseas y los vómitos inducidos por la quimioterapia y posteriormente se adaptó para medir la gravedad de las náuseas y los vómitos durante el embarazo^(16)^. Basándose en el sistema de puntuación de Rhodes, el PUQE incluye tres ítems que evalúan la duración de las náuseas, el número de episodios de vómitos y el número de episodios de arcadas. La puntuación total oscila entre 3 y 15, donde los valores de 3 a 6 indican náuseas y vómitos leves, de 7 a 12 moderados y de 13 a 15 graves^(14,16,17)^. Estudios previos han demostrado una fuerte correlación entre el PUQE y el Índice de Rhodes original. Además, Sucu et al. (2009) informaron que, al determinar la necesidad de hospitalización, el PUQE demostró una sensibilidad del 94,74 %, una especificidad del 85,71 %, un valor predictivo positivo del 72 % y un valor predictivo negativo del 97,67 % en comparación con el examen físico^(14)^.

Estos hallazgos confirman que el PUQE es un instrumento válido y confiable para evaluar la gravedad de las náuseas y los vómitos durante el embarazo.

*Escala de Evaluación de la Salud Psicosocial durante el Embarazo (PPHAS):* La PPHAS, desarrollada por Yıldız (2011), es un instrumento validado y confiable diseñado para evaluar el bienestar psicosocial durante el embarazo^(15)^. La escala consta de 46 ítems calificados en un formato tipo Likert de 5 puntos e incluye seis subescalas: Embarazo y relación conyugal (13 ítems), Ansiedad y estrés (8 ítems), Violencia doméstica (8 ítems), Necesidad de apoyo psicosocial (7 ítems), Características familiares (4 ítems) y Cambios físico- psicosociales relacionados con el embarazo (6 ítems). Las puntuaciones totales oscilan entre 46 y 230. La puntuación media, obtenida al dividir la puntuación total por el número de ítems, oscila entre 1 y 5, donde los valores de 1,00–1,79 indican «muy deficiente», 1,80–2,59 «deficiente», 2,60–3,39 «moderada», 3,40–4,19 «buena» y 4,20–5,00 «muy buena» salud psicosocial. En el estudio original, la escala mostró un coeficiente alfa de Cronbach de 0,93,12 mientras que, en el presente estudio, la consistencia interna se mantuvo alta con un alfa de Cronbach de 0,91.

Durante la entrevista inicial, las participantes completaron el Formulario de Información Descriptiva del Embarazo, el PUQE y el PPHAS. Tras completar los cuestionarios, las participantes recibieron instrucciones individualizadas sobre estrategias para controlar las náuseas y recibieron capacitación sobre el uso correcto de las pulseras.

Se indicó a las participantes que usaran las pulseras en casa durante cinco días consecutivos y que completaran el PUQE cada noche a las 10:00 p. m. El quinto día, también se les pidió que completaran el PPHAS una vez más. A lo largo de la fase en el hogar, los investigadores mantuvieron una comunicación en línea regular, que incluía información, recordatorios y apoyo de consulta.

El quinto día de la intervención, se realizaron entrevistas en línea de aproximadamente 10 a 15 minutos de duración con las participantes. Durante estas sesiones, los participantes compartieron los resultados de los formularios de evaluación que habían completado en casa, y los datos se registraron en formularios de seguimiento de casos individuales. Además, se recopilaron las opiniones, sugerencias y preguntas de los participantes con respecto a la intervención para proporcionar información complementaria sobre su eficacia percibida.

### Análisis y Tratamiento de Datos

Los datos se analizaron utilizando IBM SPSS Statistics versión 25,0. En primer lugar, se utilizaron estadísticas descriptivas para resumir las características sociodemográficas y obstétricas de los participantes. Se aplicó la prueba de chi- cuadrado (*χ^2^*) para examinar si los grupos de intervención y control eran comparables en estas características. La normalidad de la distribución de los datos se evaluó mediante las pruebas de Kolmogorov-Smirnov y Shapiro-Wilk, las cuales indicaron que los datos seguían una distribución normal. Las diferencias entre grupos en las puntuaciones medias se evaluaron mediante pruebas t para muestras independientes, mientras que los cambios dentro de los grupos a lo largo del tiempo se analizaron mediante un ANOVA de medidas repetidas. Cuando el análisis de medidas repetidas reveló un efecto significativo, se realizaron comparaciones por pares post hoc utilizando la corrección de Bonferroni para identificar los momentos específicos en los que se produjeron las diferencias. Se consideró estadísticamente significativo un p-valor inferior a 0,05 en todos los análisis.

### Aspectos éticos

Se obtuvo la aprobación ética para el estudio del Comité de Ética del Hospital de Formación e Investigación Zeynep Kamil para las Enfermedades de la Mujer y del Niño (con fecha del 06,05,2020, n.º 86).

Los centros de salud donde se llevó a cabo la investigación también otorgaron los permisos institucionales necesarios. Se informó a todos los participantes, tanto verbalmente como por escrito, sobre el propósito del estudio, los procedimientos y sus derechos como participantes. Se obtuvo el consentimiento informado por escrito de cada participante antes de su inclusión en el estudio.

### Limitaciones

Aunque el tamaño de la muestra en este estudio es suficiente según los resultados del análisis de poder, la generalización de los hallazgos es limitada debido a su tamaño relativamente pequeño. Por lo tanto, se recomiendan estudios adicionales con muestras más grandes y diferentes.

## RESULTADOS

Los grupos de la pulsera de acupresión y la pulsera placebo fueron comparables en términos de características sociodemográficas, sin diferencias estadísticamente significativas entre los grupos (p > 0,05) (Tabla 1>).

**Tabla 1:** Distribución de los participantes por características demográficas y de salud – Estambul, Turquía, 2021-2022.

Características	Pulsera de acupresión (n:31)		Pulsera placebo (n:30)		*χ^2^*	p*
	n	%	n	%		
Edad (años) media ± DE (min-máx)	27,4 ± 5,8 (20–39)		28,3 ± 5,5 (21–42)		17,688	0,409
Nivel educativo						
Primaria	3	9,7	4	13,3	7,726	0,102
Secundaria	11	35,5	4	13,3		
Bachillerato	9	29,0	11	36,7		
Licenciatura	8	25,8	11	36,7		
Situación laboral						
Con empleo	7	22,6	10	33,3	0,877	0,258
Sin empleo	24	77,4	20	66,7		
Seguridad social						
Sí	28	90,3	29	96,7	1,001	0,319
No	3	9,7	1	3,3		
Estructura familiar						
Nuclear	28	90,3	30	100,0	3,53	0,125
Extendida	3	9,7	0	0,0		
Enfermedad crónica						
Sí	9	2,0	7	23,3	0,256	0,416
No	22	71,0	23	76,7		
Fumador						
Sí	5	16,1	6	20,0	1,581	0,454
No	26	83,9	24	80,0		

Prueba de chi cuadrado *χ^2^*

*p < 0,05.

Los grupos de la pulsera de acupresión y la pulsera placebo mostraron características obstétricas similares, sin diferencias estadísticamente significativas entre ellos (p > 0,05) (Tabla 2).

**Tabla 2:** Distribución de las participantes según características obstétricas – Estambul, Turquía, 2021-2022.

Características obstétricas	Pulsera de acupresión (n:31)		Pulsera placebo (n:30)		*χ^2^*	p*
	n	%	n	%		
Gravidez media ± DE (min-max)	2,1 ± 1,42 (1–7)		2,6 ± 1,37 (1–6)		6,045	0,355
Semana de gestación media ± DE (min-max)	13,3 ± 1,42 (8–15)		12,8 ± 1,42 (7–15)		4,442	0,265
Número de hijos vivos						
0	17	54,8	14	46,7	1,994	0,737
1	9	29,0	10	33,3		
2	3	8,7	4	13,3		
3	2	6,5	2	6,7		
Paridad						
Nulípara	12	38,7	11	36,7	0,075	0,524
Multípara	19	61,3	19	63,3		
Intención de embarazo						
Intencional	25	80,6	25	83,3	3,985	0,245
No intencional	6	19,4	5	16,7		
Antecedentes de náuseas y vómitos en embarazos anteriores						
Si	14	45,2	13	43,3	2,608	0,271
No	5	16,1	6	20,0		
Primer embarazo	12	38,7	11	36,7		
Antecedentes familiares de náuseas y vómitos					1,761	0,164
Si	24	77,4	27	90,0		
No	7	22,6	3	10,0		
Conocimientos sobre el embarazo, el parto y el posparto						
Adecuada	15	48,4	20	66,7	2,069	0,118
Inadecuada	16	51,6	10	33,3		
Participación en programas de educación prenatal						
Si	6	19,4	8	26,7	4,546	0,135
No	25	80,6	22	73,3		
Atención prenatal regular						
Si	29	93,5	27	90,0	1,816	0,172
No	2	6,4	3	10,0		

*χ^2^* chi square test

*p < 0,05.

La Tabla 3 presenta la comparación de las puntuaciones del PUQE entre el grupo de la pulsera de acupresión y el grupo de la pulsera placebo. Antes de la intervención, la puntuación media del PUQE fue de 9,65 ± 2,35 en el grupo de la pulsera de acupresión y de 9,62 ± 2,20 en el grupo de la pulsera placebo, sin que se observara una diferencia estadísticamente significativa (p = 0,059). Tras la intervención, la puntuación media en el grupo de la pulsera de acupresión disminuyó a 5,26 ± 2,14, mientras que el grupo de la pulsera placebo tuvo una puntuación media de 7,86 ± 2,37. Esta diferencia entre los grupos fue estadísticamente significativa (p = 0,003).

**Tabla 3:** Comparación de las puntuaciones del PUQE intragrupales en mujeres embarazadas – Estambul, Turquía, 2021-2022.

Grupos	X ± DE	p
Pre Intervención		
Pulsera de acupresión	9,65 ± 2,35	0,059*
Pulsera placebo	9,62 ± 2,20	
Post Intervención		
Pulsera de acupresión	5,26 ± 2,14	0,003*
Pulsera placebo	7,86 ± 2,37	

Prueba t para muestras independientes

*p < 0,05.

La Tabla 4 presenta la distribución de la gravedad de las náuseas y los vómitos (puntuaciones PUQE) en las mujeres embarazadas a lo largo de la intervención. En el grupo de la pulsera de acupresión, la puntuación media PUQE fue de 9,65 ± 2,35 al inicio del estudio y disminuyó desde el primer día: 7,93 ± 1,50 el primer día, 5,80 ± 1,77 el segundo día, 5,67 ± 2,44 el tercer día, 5,34 ± 2,18 el cuarto día y 5,26 ± 2,14 el quinto día. El análisis de medidas repetidas confirmó que esta reducción fue estadísticamente significativa (p < 0,001). Las comparaciones post hoc indicaron que las diferencias más significativas se produjeron entre la medición inicial y los días dos a cinco. Por el contrario, el grupo de la pulsera placebo mostró solo una ligera tendencia a la baja en las puntuaciones medias del PUQE a lo largo de los cinco días (9,62 ± 2,20 en la línea de base; 7,86 ± 2,37 en el día cinco), lo cual no fue estadísticamente significativo (p > 0,05). Estos resultados sugieren que la aplicación de la pulsera de acupresión reduce eficazmente la gravedad de las náuseas y los vómitos en las mujeres embarazadas, mientras que la pulsera placebo no produce un efecto comparable.

**Tabla 4:** Comparación de las puntuaciones del PUQE entre los grupos experimental y de control – Estambul, Turquía, 2021-2022.

Grupos	Proceso de intervención	X ± DE	p*
Pulsera de acupresión	Inicial	9,65 ± 2,35	0,000*
	Día 1	7,93 ± 1,50	
	Día 2	5,80 ± 1,77	
	Día 3	5,67 ± 2,44	
	Día 4	5,34 ± 2,18	
	Día 5	5,26 ± 2,14	
Pulsera placebo	Inicial	9,62 ± 2,20	0,063
	Día 1	9,36 ± 2,20	
	Día 2	9,10 ± 1,90	
	Día 3	8,46 ± 2,11	
	Día 4	8,10 ± 1,98	
	Día 5	7,86 ± 2,37	

Prueba ANOVA

*p < 0,05.

La Tabla 5 presenta la comparación de las puntuaciones del PPHAS entre los grupos de la pulsera de acupresión y la pulsera placebo a lo largo de la intervención. Antes de la intervención, la puntuación media del PPHAS fue de 2,94 ± 0,27 en el grupo de la pulsera de acupresión y de 2,99 ± 0,31 en el grupo placebo, sin diferencias estadísticamente significativas entre los grupos (p = 0,746). Después de la intervención, las puntuaciones medias aumentaron a 3,22 ± 0,24 en el grupo de la pulsera de acupresión y a 3,16 ± 0,29 en el grupo de la pulsera placebo; sin embargo, esta diferencia siguió sin ser estadísticamente significativa (p = 0,355). Sin embargo, las comparaciones intragrupales revelaron una mejora significativa en ambos grupos desde antes hasta después de la intervención (grupo de acupresión, p = 0,000; grupo de placebo, p = 0,000). Estos hallazgos sugieren que, si bien ambas intervenciones con pulseras se asociaron con un mayor bienestar psicosocial en las mujeres embarazadas, la adición de la acupresión no produjo un efecto estadísticamente mayor en comparación con el placebo.

**Tabla 5:** Comparación de las puntuaciones del PPHAS – Estambul, Turquía, 2021-2022.

Grupos		X ± DE	p*
Pre- Intervention	Pulsera de acupresión	2,94 ± 0,27	0,746
	Pulsera placebo	2,99 ± 0,31	
Post-Intervention	Pulsera de acupresión	3,22 ± 0,24	0,355
	Pulsera placebo	3,16 ± 0,29	
Pulsera de acupresión	Pre Intervención	2,94 ± 0,27	0,000*
	Post Intervención	3,22 ± 0,24	
Pulsera placebo	Pre Intervención	2,99 ± 0,31	0,000*
	Post Intervención	3,16 ± 0,29	

PPHAS: Escala de evaluación de la salud psicosocial durante el embarazo, prueba t para muestras independientes, prueba ANOVA

*p < 0,05.

## DISCUSIÓN

Este estudio analizó la eficacia de la acupresión aplicada en el punto P6 para reducir las náuseas y los vómitos durante el embarazo, así como su posible impacto en la salud psicosocial. Las náuseas y los vómitos relacionados con el embarazo suelen estar asociados a niveles elevados de gonadotropina coriónica humana beta (β-hCG). Dados los posibles riesgos de los tratamientos farmacológicos para el feto, los enfoques no farmacológicos son cada vez más preferidos para el manejo de los síntomas^(11)^. La acupresión representa uno de esos métodos seguros y no invasivos. La evidencia de la literatura indica que la estimulación de puntos específicos de acupresión puede aumentar los niveles de β-endorfinas, y que concentraciones más altas de endorfinas y 5-hidroxitriptamina (5-HT) pueden ayudar a aliviar los síntomas de náuseas y vómitos^(7)^. Los estudios que investigan la eficacia de la acupresión para el manejo de las náuseas y los vómitos durante el embarazo varían en cuanto a los grupos de comparación, la duración de la intervención y los períodos de seguimiento. Un estudio, por ejemplo, comparó grupos de acupresión, placebo y control. En un estudio realizado en Irán, el grupo experimental recibió acupresión en el punto P6 utilizando el botón Sea-Band durante tres días, el grupo placebo usó el Sea-Band sin aplicar presión en el punto P6, y el grupo de control recibió solo recomendaciones dietéticas, similares a las dadas a los otros dos grupos. La frecuencia y la gravedad de las náuseas y los vómitos se evaluaron dos veces al día durante seis días, a partir del cuarto día de la intervención. Después de tres días, tanto el grupo de acupresión como el de placebo mostraron una reducción significativa en la frecuencia, la duración y la gravedad de las náuseas, así como en la frecuencia de los vómitos, mientras que no se observó tal reducción en el grupo de control^(12)^. En otro estudio, no se administró ningún tratamiento a ningún grupo durante los primeros tres días; posteriormente, se aplicó acupresión en el punto P6 al grupo experimental durante un período de cuatro días. Aunque no se observaron diferencias significativas entre los grupos de acupresión y placebo en cuanto a las náuseas y los vómitos, ambos grupos experimentaron niveles de síntomas significativamente más bajos en comparación con el grupo de control^(18)^. En estos estudios, la gravedad de las náuseas y los vómitos no varió entre los participantes que recibieron acupresión real y los que recibieron placebo, lo que sugiere que, más allá de los efectos fisiológicos de la acupresión, la creencia de los pacientes en la intervención y el acto de la estimulación táctil en sí mismo pueden producir un efecto terapéutico significativo. En otro tipo de estudio, se comparó la eficacia de la acupresión con los métodos de tratamiento estándar. En un estudio realizado en Malasia, el grupo experimental recibió acupresión en el punto P6 tres veces al día durante al menos 10 minutos, además de líquidos intravenosos y el antiemético de rescate metoclopramida (10 mg). El grupo de control recibió líquidos intravenosos y metoclopramida (10 mg) cada 8 horas durante 24 horas. Los resultados indicaron que el grupo experimental experimentó una reducción de las náuseas y los vómitos, y su necesidad de antieméticos adicionales fue significativamente menor que la del grupo de control. El estudio demuestra que, cuando se aplica junto con la atención estándar, la acupresión tiene un efecto significativo en la reducción de las náuseas y los vómitos^(8)^. En otro estudio, se comparó la eficacia de la acupresión entre los grupos de acupresión, placebo y tratamiento estándar. Realizado en Irán, el estudio asignó a un grupo a recibir acupresión en el punto P6 cuatro veces al día durante 10 minutos, a un segundo grupo a recibir acupresión simulada (placebo) y a un tercer grupo a recibir vitamina B6 y metoclopramida. Las evaluaciones realizadas antes de la intervención, así como el primer y el quinto día después de la intervención, revelaron que, para el quinto día, surgieron diferencias significativas en la frecuencia de los vómitos, la angustia por las arcadas, la angustia por los vómitos, la duración de las náuseas, la angustia por las náuseas, el volumen de los vómitos, la frecuencia de las náuseas y la frecuencia de las arcadas. Estos hallazgos sugieren que la estimulación del punto de acupresión P6 puede reducir eficazmente la gravedad de las náuseas, los vómitos y las arcadas en las mujeres embarazadas^(13)^. En otra serie de estudios, se compararon los resultados entre los grupos de acupresión y los grupos de control. En Turquía, un estudio asignó al grupo experimental a recibir acupresión en el punto P6 tres veces al día durante una semana, mientras que el grupo de control no recibió ninguna intervención. Los resultados indicaron una reducción significativa de las náuseas y los vómitos en el grupo de acupresión^(13,19)^. De manera similar, en otro estudio, el grupo experimental recibió acupresión durante una semana, mientras que el grupo de control no recibió ningún método para aliviar las náuseas y los vómitos; una vez más, la acupresión redujo significativamente las puntuaciones de náuseas y vómitos, mientras que no se observaron cambios notables en el grupo de control^(11)^. Sin embargo, los resultados de estos dos estudios realizados en Turquía revelan cierta variabilidad en cuanto a la eficacia de la acupresión. Otro tipo de estudio se centró en comparar la acupresión con un placebo. En uno de estos estudios, el grupo experimental recibió acupresión en el punto P6 durante aproximadamente 60 minutos diarios durante siete días consecutivos, mientras que el grupo de control recibió solo contacto en el punto P6, siguiendo el mismo procedimiento y duración, sin presión activa. En el grupo experimental, tanto la gravedad como la frecuencia de las náuseas y los vómitos se redujeron significativamente tras el inicio del tratamiento en comparación con el grupo de placebo^(20)^. El estudio concluyó que la acupresión en el punto P6 fue más eficaz para aliviar las náuseas y los vómitos que la intervención con placebo. La evidencia de la literatura respalda aún más estos hallazgos. Dos metaanálisis han evaluado la eficacia de la acupresión para las náuseas y los vómitos relacionados con el embarazo. En un metaanálisis, que incluyó 11 ensayos controlados aleatorios con un total de 1378 mujeres embarazadas, la acupresión se asoció con mayores mejoras en los síntomas de náuseas y vómitos en comparación con los grupos de placebo y de control. Además, la acupresión demostró beneficios en la prevención de las náuseas y los vómitos, la reducción de la duración de la estadía hospitalaria y la mejora de la satisfacción de las pacientes en relación con el placebo^(6)^. Otro metaanálisis, que incluyó 33 estudios con 3,390 mujeres embarazadas, encontró de manera similar que la acupresión redujo significativamente las puntuaciones de náuseas, acortó la estancia hospitalaria y mejoró la calidad de vida general^(7)^.

Los hallazgos de este estudio son consistentes con los reportados en la literatura, lo que indica que la estimulación del punto de acupresión P6 puede aliviar eficazmente las náuseas y los vómitos durante el embarazo. Sin embargo, algunos estudios no han encontrado diferencias significativas entre la acupresión y las intervenciones simuladas (placebo), lo que sugiere que los beneficios de la acupresión pueden derivarse no solo de mecanismos fisiológicos, sino también de efectos psicológicos o placebo. Las diferencias en el diseño del estudio, la duración y la frecuencia de la intervención, el momento de las evaluaciones de los resultados y el contexto cultural pueden explicar las inconsistencias entre los estudios. En conjunto, la evidencia meta-analítica respalda en general el uso de la acupresión como enfoque complementario para el manejo de las náuseas y los vómitos relacionados con el embarazo; sin embargo, estos hallazgos resaltan la necesidad de una estandarización metodológica y de ensayos controlados aleatorios más rigurosos que comparen directamente la acupresión con intervenciones simuladas (placebo).

Las mujeres embarazadas que experimentan náuseas y vómitos a menudo reportan niveles elevados de estrés, ansiedad, depresión y preocupaciones sobre posibles daños a sus bebés^(21,22)^. Sin embargo, los estudios que examinan los efectos de la acupresión sobre el bienestar psicosocial en esta población son limitados. En un estudio, se aplicó acupresión en el punto P6 para aliviar las náuseas y los vómitos y en el punto L14 para reducir la ansiedad, durante 15 minutos en cada caso, diariamente durante siete mañanas consecutivas al despertar. Ambos grupos de intervención recibieron el mismo protocolo de acupresión, mientras que el grupo de control no recibió ninguna intervención. El estudio encontró que la acupresión en los puntos P6 y L14 redujo eficazmente la ansiedad^(23)^. En el presente estudio, el bienestar psicosocial mejoró tanto en el grupo de acupresión como en el de control; sin embargo, no se observó una diferencia estadísticamente significativa entre los grupos. Estos hallazgos sugieren que el impacto de la acupresión en la salud psicosocial puede verse influido por factores como la variabilidad individual, la duración y la ubicación de la aplicación, el momento de la evaluación o los efectos placebo. Se necesitan más investigaciones para aclarar el papel de la acupresión en la mejora del bienestar psicosocial entre las mujeres embarazadas.

El estudio se llevó a cabo en un solo centro con un tamaño de muestra relativamente pequeño, lo que limita la generalización de los hallazgos. No se evaluaron los efectos a largo plazo de la intervención. Además, el aumento del bienestar psicosocial observado en el grupo de control sugiere que no se pudieron descartar por completo los efectos placebo. Las investigaciones futuras deberían tener como objetivo confirmar estos hallazgos mediante ensayos controlados aleatorios más amplios, multicéntricos y a largo plazo.

## CONCLUSIÓN

Este estudio evaluó el efecto de la acupresión aplicada en el punto P6 sobre la gravedad de las náuseas y los vómitos y sobre el bienestar psicosocial en mujeres embarazadas. Los resultados indican que la acupresión es eficaz para aliviar los síntomas de náuseas y vómitos durante el embarazo. Aunque se observaron mejoras en la salud psicosocial tras la aplicación de la acupresión, estos cambios no fueron estadísticamente significativos en comparación con el grupo de control. Estos resultados sugieren que la acupresión puede contribuir al bienestar psicosocial además de proporcionar alivio físico, pero el efecto parece ser limitado. Dada su seguridad, su carácter no invasivo, su bajo costo y su facilidad de aplicación, la acupresión puede considerarse un método práctico para el manejo de las náuseas y los vómitos relacionados con el embarazo. La incorporación de la acupresión en un enfoque holístico de la atención de enfermería puede apoyar tanto el manejo de los síntomas como las necesidades psicosociales de las mujeres embarazadas. Se necesitan más ensayos clínicos a gran escala y de alta calidad para confirmar su eficacia y sus beneficios a largo plazo.

## Data Availability

El conjunto de datos completo en el que se basan los resultados de este estudio está disponible previa solicitud al autor correspondiente.
